# A synthesis of recent tools and perspectives in migratory connectivity studies

**DOI:** 10.1186/s40462-023-00388-z

**Published:** 2023-10-27

**Authors:** Killian A. Gregory, Charlotte Francesiaz, Frédéric Jiguet, Aurélien Besnard

**Affiliations:** 1 Master de Biologie, École Normale Supérieure de Lyon, Université Claude Bernard Lyon 1, Université de Lyon, Lyon, France; 2 CESCO, MNHN-CNRS-Sorbonne Université, Paris, France; 3 CEFE, Univ Montpellier, CNRS, EPHE, IRD, Montpellier, France; 4 OFB, DRAS, Juvignac, France; 5 CEFE, Univ Montpellier, CNRS, EPHE-PSL University, IRD, Montpellier, France

**Keywords:** Migration patterns, Transition probabilities, Data pooling, Integrated modelling, Population dynamics, Movement ecology

## Abstract

Migration movements connect breeding and non-breeding bird populations over the year. Such links, referred to as migratory connectivity, have important implications for migratory population dynamics as they dictate the consequences of localised events for the whole population network. This calls for concerted efforts to understand migration processes for large-scale conservation. Over the last 20 years, the toolbox to investigate connectivity patterns has expanded and studies now consider migratory connectivity over a broader range of species and contexts. Here, we summarise recent developments in analysing migratory connectivity, focusing on strategies and challenges to pooling various types of data to both optimise and broaden the scope of connectivity studies. We find that the different approaches used to investigate migratory connectivity still have complementary strengths and weaknesses, whether in terms of cost, spatial and temporal resolution, or challenges in obtaining large sample sizes or connectivity estimates. Certain recent developments offer particularly promising prospects: robust quantitative models for banding data, improved precision of geolocators and accessibility of telemetry tracking systems, and increasingly precise probabilistic assignments based on genomic markers or large-scale isoscapes. In parallel, studies have proposed various ways to combine the information of different datasets, from simply comparing the connectivity patterns they draw to formally integrating their analyses. Such data combinations have proven to be more accurate in estimating connectivity patterns, particularly for integrated approaches that offer promising flexibility. Given the diversity of available tools, future studies would benefit from a rigorous comparative evaluation of the different methodologies to guide data collection to complete migration atlases: where and when should data be collected during the migratory cycle to best describe connectivity patterns? Which data are most favourable to combine, and under what conditions? Are there methods for combining data that are better than others? Can combination methods be improved by adjusting the contribution of the various data in the models? How can we fully integrate connectivity with demographic and environmental data? Data integration shows strong potential to deepen our understanding of migratory connectivity as a dynamic ecological process, especially if the gaps can be bridged between connectivity, population and environmental models.

**Supplementary Information:**

The online version contains supplementary material available at 10.1186/s40462-023-00388-z.

## Introduction

Migratory birds move seasonally between breeding and non-breeding sites in response to environmental changes and genetic cues [1]. In visiting multiple areas across seasons, they encounter various pressures including unpredictable environmental conditions and resource availability, hunting and predation risks, and anthropogenic degradation of migratory routes. Combined with the energetic challenges of such a long journey, these pressures make long-distance migration particularly risky [2]. In fact, it has been shown that both habitat loss during the breeding season, and anthropogenic and climatic factors during the non-breeding season, are the main causes of large-scale declines in long-distance Afro-Palaearctic migrants since the 1970s [2, 3]. In addition, lower survival has been described for some migratory birds during migratory periods ([4, 5]; but see [6]). Yet pressures happening during the breeding, migration, and non-breeding periods do not solely result in a direct increase in mortality. Events occurring at one stage of the migratory cycle are also known to influence the breeding success or survival of individuals in subsequent stages, a process known as seasonal interaction [7]. Thus, a clearer understanding of the ecology, spatial dynamics, and demography of migratory species requires deciphering the links between populations at different stages of the migration cycle. Such links, drawn by the periodic movements of migratory individuals between populations or areas, have been defined as migratory connectivity [8].

The importance of migratory connectivity for conservation biology has been emphasised from the very beginning, and has found support in a diversity of studies since then [8], becoming a cornerstone for international initiatives such as the Convention on the Conservation of Migratory Species of Wild Animals [9]. Beyond bringing the long-awaited data enabling the population dynamics of different seasons to be connected [10], migratory connectivity is potentially instrumental in the transmission of pathogens [11]. It can also help correlate localised environmental changes or anthropogenic pressures and distant population declines [12, 13], understand the robustness of migration networks (e.g. [14, 15]), and contribute to identifying conservation hotspots. Migratory connectivity influences how the spatial structuring of populations and the movement of individuals may affect species susceptibility to global changes [8, 16]. It is thus among the main priorities for research to devise successful conservation measures for migratory species [17–19].

Multiple methods have been used to identify the connections between the breeding and wintering grounds of migratory birds [8]. Bird banding has been used since the beginning of the twentieth century to track individuals between their marking and reencounter sites [20]. Stable isotopes and genetics have been used to study migratory connectivity more recently [21–23]. Such markers, referred to as “intrinsic markers”, are naturally carried by every individual and can provide information on the geographic origin of the individual without needing to mark it. In parallel, the development and miniaturisation of tracking devices such as radio and satellite transmitters, geolocators, and the establishment of large-scale telemetry networks such as MOTUS [24] and the Icarus initiative [25], have enabled explicit spatial data about migration tracks to be collected [26, 27].

**Fig. 1:**
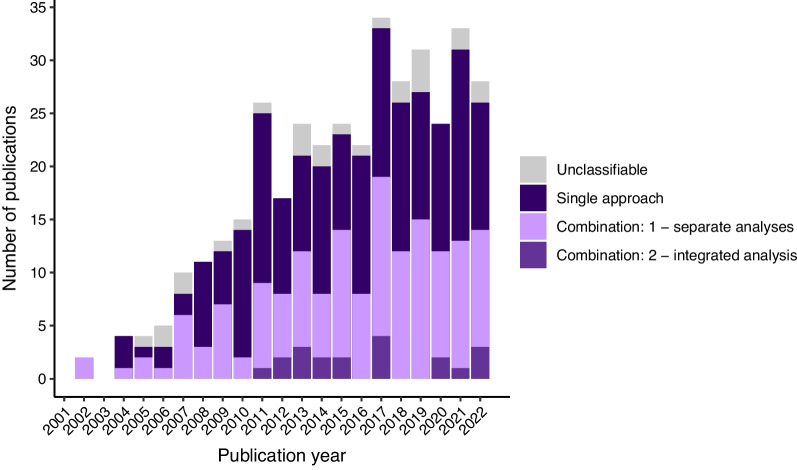
Number of peer-reviewed publications about migratory connectivity in the Web of Science Core Collection. The number of publications mentioning only one approach (dark purple), or data from at least two different types of approach either analysed separately (light purple) or integrated in a single framework (medium purple) are shown by year. Publications were selected based on the occurrence of selected word patterns in the title, abstract or keywords (search conducted on 02/11/2023; see Additional file 1). 7% of publications could not be classified using our methodology (grey). The absolute numbers are expected to be underestimated, but the relative proportions of analysis types are assumed to be representative. Data from Web of Science, provided by Clarivate. Web of Science and Clarivate are trademarks of their respective owners and used herein with permission

The continuous improvement of these key approaches over the last 20 years has led to a significant increase in the number of publications on migratory connectivity (Fig. 1). The expanding toolbox of approaches to investigate migratory connectivity has allowed these studies to diversify and start investigating both fundamental questions about the establishment and consequences of connectivity patterns (e.g. [28]) and applied questions about species conservation and epidemiology (e.g. [29]). Additionally, the growing emphasis on data integration in ecology has fuelled this trend, with the potential to offer a major turning point in broadening our perception of migratory connectivity by allowing the construction of direct bridges between connectivity and population or environmental studies. Indeed, data integration appears to be a promising tool for bringing together what are currently considered to be separate fields of biology [30].

With the multiplication of studies investigating connectivity patterns along with en-route behaviour (e.g. [31]), environmental changes (e.g. [14]), or population dynamics (e.g. [15]), the field of migratory connectivity has never been as close to other ecological fields as it is now. Yet if promising applications have already highlighted the potential of connectivity for future research, much of the data remains to be collected. We believe that, for the field to fully contribute to answering broader ecological questions, transversal studies are needed, which may benefit from making the methodologies to investigate migratory connectivity more accessible. Here, we provide an overview of the latest advances to investigate migratory connectivity and discuss how these methodologies are developing. A particular focus is the promise, as well as the challenges, of data integration, a topic that will become increasingly important as migratory connectivity is applied in a wider range of contexts.

To establish this synthesis, we scanned through the literature returned by Google Scholar and the Web of Science using the broad search pattern “migrat* connectivity”, in combination with a series of keywords referring to the various types of data and data combinations used in the studies (see Additional file 1). We could then focus on (1) articles pointed out by several other publications as establishing major advances in each approach, in order to identify the most recent methodological developments; (2) major articles and reviews giving extensive information on each individual approach for further information; and (3) articles using a combination of data to investigate connectivity patterns, in order to build up a picture of the existing combinations and their use.

## Tools to investigate migratory connectivity

### Bird banding

Traditionally, birds captured on their breeding or non-breeding grounds have been marked with leg bands or other markers, indicating their place of origin if they are later recaptured, resighted, or their corpse is recovered elsewhere [20]. While banding data has long been used to draw qualitative connectivity patterns, it is now the main data allowing quantitative links between populations to be estimated (Box 1).

**Box 1:** What description of migratory connectivity?

Studies tackling migratory connectivity issues revolve around at least one out of three features that describe the movements of individuals. Concepts are described below for spring migration (movements from breeding to non-breeding/wintering sites), but they are equally applicable to autumn migration (movements from non-breeding/wintering to breeding sites).		
**(i) Connectivity patterns:**		
• What?	Qualitative description of links between populations or sites at different stages of the migratory cycle. Patterns can be further described based on how the links are arranged in relation to each other (“longitudinal”/ “parallel”/ “leap-frog”/ “cross-wise” migration; see [55] for a review).	
• How?	Generally based solely on the spatial assignment of samples, individuals or groups of individuals to their location in the previous season.	
• What data?	All approaches used to track individuals through space (banding, tracking technologies, genetic and isotopic assignments, e.g. [52, 56]).	
**(ii) Transition probabilities**		
• What?	Probability that an individual of population A moves to population B. Transition probabilities can be thought of as a quantitative description of links between populations, thus giving a direct estimation of migratory connectivity if estimated between breeding and wintering populations [57]. This concept ties the population-level metrics of migratory connectivity with individual-level behaviour.	
• How?	Relies on models that are able to account for biases due to uneven sampling that obscure the true transition probabilities [12, 32, 57].	
• What data?	Such models have mostly been developed for banding data [32, 34], and more recently tracking [12, 57] and isotope data [40].	
**(iii) Connectivity strength**		
• What?	A metrics describing how much the structuring of populations in one season is conserved in the next season [8]. There is a continuum between a complete mixing of previously isolated populations (“weak” or “diffuse” connectivity) and the conservation of the same structure between both seasons (“strong” connectivity), which depends on both population spread and inter-population mixing [16]. This property of the migratory system is likely to have an influence on the susceptibility of species to habitat loss, climate change, and population declines [16].	
• How?	Two metrics have been adapted or developed to estimate connectivity strength: - Mantel test quantifies the correlation between matrices of inter-individual distances (usually one for the breeding and one for the non-breeding season; [58]). - MC completes the Mantel test by including transition probabilities and taking biases in data collection into account [59].	
• What data?	Usually performed on banding or tracking data that locates individuals with good spatial resolution, but recently adapted to include isotopic data [60, 61]. MC is notably under constant improvement to integrate more diverse data.	

This shift from qualitative patterns to quantified estimates of migratory connectivity relies on statistical models capable of disentangling the probability of movement between populations (i.e. connectivity parameters) from survival probability and spatial variation in banding effort and reencounter probability, which are intertwined in raw data [32]. These probabilities serve to express the general probability of reencountering a marked bird in a given area, depending on the type of available data—live reencounter or dead recovery—in multi-state mark–reencounter models used to investigate the movement of individuals between predefined states (i.e. sites and/or seasons in this case) [32–35]. The division coefficient method offers a straightforward alternative to quantify migratory connectivity over long time periods by estimating movement probabilities between sites [36]. The aim of this method is first to estimate reencounter probabilities in each destination from simple systems of equation, assuming that these probabilities are the same whatever the origin of the individuals. Knowing the number of birds ringed in each source area, the number of birds reencountered in each destination and the reencounter probabilities then enables the calculation of the movement probabilities.

Recent methodological developments exploit the flexibility of mark–reencounter modelling frameworks to deal with the spatial and temporal heterogeneity of banding data, which stems from the unequal number of observers or observations between seasons and places (Table 1A; see [37, 38], for a review). These recent approaches can take into account sources of heterogeneity such as seasonal variation in reencounter probability [34] or can integrate new information known to affect reencounter probability such as human density in the recovery region [39]. Models have also even been improved to work when information on the total banding effort is lacking [35, 39, 40]. Overall, these developments have been carried out using Bayesian modeling, which is particularly flexible for building complex models that notably take various sources of uncertainty into account and allow for the combination of various types of banding data, favouring numeric estimations (e.g. [34, 35]).

**Table 1:** Intrinsic advantages and drawbacks of the four main approaches to study migratory connectivity

Approach/data	Main advantages	Main drawbacks	Developments^a^	References
**A. Bird banding**	• ﻿Huge datasets • ﻿Inexpensive • ﻿Explicit information of marking origin • ﻿Quantification of links between populations	• ﻿Sometimes unknown number of banded birds (**MS**) • ﻿Survival bias (**MS**) • ﻿Recapture and recovery spatial and temporal heterogeneity (**MS**) • ﻿Low probability of recovery • ﻿Advanced models to become quantitative (**DC**)	***MS****: Multi-state models* ***DC****: Division coefficient*	[34–38]
**B. Tracking technologies**	• ﻿Explicit data on the whole migratory route	• ﻿Cost (limited number of tagged individuals) • ﻿Weight (constraints on equippable species, effects on behaviour/survival)		
*> Radio telemetry*	• ﻿Cheap • ﻿Light (< 1 g)	• ﻿Proximity of receiver station needed (**RN**)	***RN****: Receiver networks (Motus Wildlife Tracking System)*	[24]
*> Archival geolocators*	• ﻿Cheap • ﻿Light (< 2 g)	• ﻿Low spatial resolution (**TA**, **SM**) • ﻿Recovery needed (survival and recapture bias) (**RM**)	***TA****: Technical advances (e.g. light stalks, archival GPS tags)* ***SM****: Spatial models* ***RM****: Recovery models*	[12, 41–43]
*> Satellite transmitters*	• ﻿High spatial resolution • ﻿No recovery needed	• ﻿Most expensive (**TA**) • ﻿Heaviest (2–100 g) (**TA**)	***TA****: Technical advances (e.g. miniaturisation)*	[25, 44]
**C. Genetics**	• ﻿Intrinsic marker: allows information on many birds	• ﻿Detectable genetic structure needed (dependent on recent evolutionary history) (**G**) • ﻿S﻿pecies-specific markers needed (**G**) • ﻿Low spatial resolution (**G**) • ﻿Only natal origin • ﻿C﻿ost of genome-wide analyses • ﻿Transit of biological samples for analysis	***G****: Genomic analysis*	[45, 46]
**D. Stable isotopes**	• ﻿Intrinsic marker: allows information on many birds • ﻿Inexpensive • ﻿Universal	• ﻿Extensive mapping needed • ﻿Low spatial resolution (**MI**, **PIA**, **SSM**) • ﻿Local variation sometimes higher than regional variation (e.g. elevation effect on precipitation) (**SSM**) • ﻿Inter-individual variation in discrimination (**SSM**) • ﻿Transit of biological samples for analysis	***MI****: Multi-isotope* ***PIA***:* Probabilistic isoscape-based assignments* ***SSM****: Site-specific models*	[47–49]

^a^Major developments that have succeeded in mitigating certain limitations of each approach. For details about some less common approaches to track migrants, see Hobson et al. [50]

Nevertheless, the number of reencounters generally remains very low—e.g. only two birds in Giunchi et al. [51] and barely 1,000 out of 500,000 birds tagged over 50 years in Korner-Nievergelt et al. [34]—and the spatio-temporal heterogeneity in observation effort tarnishes all available datasets [38]. Although these drawbacks can be partially offset, taken together with the complexity of the required modelling approaches, many studies remain qualitative (e.g. [52, 53]).

### Tracking technologies

Tracking technologies include electronic tags such as transponders or spatial data loggers fitted onto captured birds. Archival geolocators (e.g. [27]), satellite telemetry (e.g. [26]), and radio telemetry (e.g. [54]) are the main tools used in migratory connectivity studies. They generally provide precise and immediate information on the whole migratory route, including breeding, wintering, and stopover sites, and are thus very powerful to draw precise yet qualitative connectivity patterns—although methods to quantify migratory connectivity have recently been adapted to include scarce tracking data (Box 1).

Light-level geolocators, which derive latitude and longitude from the relative duration of day and night and the occurrence of solar noon, are widely used due to their low cost and weight [29, 47]. However, their use is limited by their particularly coarse spatial resolution (up to 300 km), which can be partly offset by the use of standardised filtering methods to remove aberrant locations, although this poses other issues in terms of reproducibility [62]. Also, the data they provide suffers from a recovery bias, since such archival tags need to be retrieved to collect the data [27]. Recent developments have aimed to increase their resolution using modelling approaches [63, 64] or through technical improvements such as the addition of light stalks that better record light intensity [41] or the conception of alternative archival GPS tags [42]. Statistical models can also be used to account for recovery bias ([12]; see [43], for a review; Table 1B).

Progressive technical advances in tracking technologies have resulted in a reduction of the cost and weight of devices—especially for satellite tags with various transmission systems (Argos, GSM or radio)—allowing more individuals and smaller species to be tagged [25, 44]. The development of continental-scale receiver networks is also likely to promote radio telemetry as a major tool for investigating migration [24] (Table 1B). The use of movement and behavioural models enables to further improve the high spatio-temporal resolution of the tracking data and obtain insights into migratory connectivity down to the scale of migratory routes [65]. And beyond telemetry, the application of machine learning to infer migratory movements from weather radar data may even enable the tracking of migratory populations on a continental scale, especially if species can be discriminated in the data [66].

Yet the cost of tracking devices remains high, leading to problems in representativeness and statistical power due to the low number of tagged individuals. As a first solution, rigorous sampling designs should help reduce bias in data collection [67], for instance by sampling individuals of the whole breeding and/or non-breeding distribution range of a species in a statistically randomised sampling design [68]. This has even been suggested as a way to reduce bias in connectivity measures [69]. However, putting the same sampling effort into remote areas as into accessible areas may add to the cost of the method and requires international collaboration. Also, the sampling design will still be limited by capture biases (i.e. which individuals are more susceptible to be captured) and monitoring biases (i.e. which individuals can be tagged). The weight of tracking devices induces a bias in our perception of migratory patterns towards larger taxa or larger individuals within a species, and attention should be paid to a potential effect on bird survival and flight performance [70].

### Genetics and genomics

Individuals using the same breeding grounds are more likely to mate and share similar genetic features. Thus, analysing the genetic diversity of samples collected on a migratory site may allow the probabilistic assignment of individuals to a reference breeding population [22] and the identification of broad connectivity patterns (Box 1).

Investigations of connectivity patterns based on genetic data have been conducted using population genetics tools on various types of DNA sequences, the most recent being mitochondrial DNA, short repeated sequences such as microsatellites, and Single-Nucleotide Polymorphism (SNPs; [8, 56, 71, 72]). All these sequences are capable of carrying information on the genetic similarity or differentiation of individuals to varying degrees. If collected on the non-breeding grounds, it is thus possible to know if two individuals come from the same breeding grounds. If reference individuals have been sampled directly on the breeding grounds and if the marker shows a geographic structure, genetic assignments can be used as geographic assignments. Therefore, an investigation of population differentiation based on discriminant analyses [29] or differentiation metrics such as the fixation index (FST) [56] can offer initial insights into the relationships between breeding and non-breeding populations. Yet most often, the breeding origin of migratory birds is determined using well-established clustering algorithms followed by one of various assignment methods (see [29, 73] for examples).

Today, technical advances enable the exploitation of genomic data [45, 73, 74]. Species-specific markers based on microsatellites or single-nucleotide polymorphisms (SNPs) can now be developed more easily [46, 72]. In this way, genomics opens the door to larger datasets capable of providing more information about the genetic structure of populations at ever-decreasing costs (Table 1C).

Nonetheless, genomic data should be handled with care, as not all parts of the genome are informative [45]. The reliable clustering of individuals requires some genetic differentiation between populations, which can be rare for animals with high dispersal ability [63]. In this case, genetic differentiation may concern only a few loci, information that can be diluted when too much irrelevant genomic data is added. Even for species having a strong genetic differentiation, identifying sequences that can be considered characteristic of a particular area or population requires extensive work to collect and analyse high-quality reference samples over the entire breeding range of the species. Additionally, despite declining costs, sequencing such a large number of samples remains costly and may pose some logistic issues due to the transport of the samples between countries. So, while genomic data has unquestionably advanced our ability to detect population differentiation and to make assignments, this approach still has inherent limitations.

### Stable isotopes

Geochemical processes result in spatial variations in stable isotope proportions within natural environments [75]. For instance, the condensation rates of heavy and light isotopes are different, leading to a spatial variation in the proportion of ^2^H and ^1^H along the clouds journey. These processes are further modulated by local variations in temperature and elevation, as well as biological processes, which paint a complex landscape of varying isotope proportions [75, 76]. These proportions are recorded in tissues such as moulting feathers [21, 23] and growing claws [77] through the uptake of food, with again some bias due to differential partitioning of light and heavy isotopes during biogeochemical processes. Individuals with similar isotopic signatures in such tissues can then be assumed to have grown them in similar isotopic environments, and thus to come from the same geographical region. Mainly used to draw large-scale connectivity patterns, isotope data is in the process of being included in quantified connectivity estimates (Box 1).

Isotopic signatures can be used to assign individuals to populations of origin using clustering tools such as discriminant analyses [78]. Knowledge of the geographical distribution of isotope values, i.e. the isoscape, further allows individuals to be directly assigned to regions using a variety of methods. These methodologies generally rely on the construction of a calibration regression model to correlate isotope values in the environment and isotope values in biological tissues of known origin (see [79], for a review). In particular, the probabilistic assignment of individuals on continuous geographical surfaces of isotope values is becoming popular ([48]; see [80], for a review).

The strength of the isotope approach is that huge datasets can be easily collected and cheaply analysed to obtain wide-scale connectivity patterns for several species [81, 82]. δ^2^H is the most common isotope ratio used to study migratory connectivity due to its clear latitudinal patterns and well-characterised isoscape described as part of long-term hydrological surveys [21, 75]. Other isotopes such as δ^13^C, δ^15^N, or δ^34^S are increasingly used to get more accurate spatial assignments when δ^2^H patterns are too weak [49, 83, 84]. Additional research has focused on means to account for inter-individual variation in fractionation and large local variation in isotope values, both of which contribute to blurring assignments ([47, 76]; see Table 1D). Applied to museum collections, this approach allows historical assignments to be performed to study variations in migratory connectivity in the face of global change [85].

Despite these developments, the isotope approach is limited by low spatial resolution, as birds are assigned to areas often spanning thousands of square kilometres (e.g. [82]). This low resolution is further amplified as probabilistic assignments only partially take into account uncertainties in measurements and models [80].

Beyond these four main approaches to investigate migratory connectivity, other methodologies have occasionally been used to extract information on animal movements between migration stages (see [50] for a more comprehensive overview). These methodologies notably include assignments based on phenotypic variation between source populations (e.g. [86]), on trace elements yielding a higher resolution than common isotopes [87], or genetic analyses performed on parasites carried by migrating individuals (e.g. [40]). Recent modelling approaches even allow accurate movements between populations to be extracted from abundance and citizen science data in some specific situations [88–90]. Given the rise of citizen science, they offer great potential to push forward and/or complement migratory connectivity studies based on more traditional approaches.

## Combining data in migratory connectivity studies

Combining different connectivity data emerged early in the 2000s as a means to buffer some of the drawbacks of each approach [49, 71, 91] (Fig. 1). A whole range of possible combinations have been used in recent studies, incorporating up to three of the previously described approaches along with complementary data (e.g. [92]) (Fig. 2.1). Combined analyses can be classified into two main families: those that pool information from different analyses performed separately, and more recent integrated approaches that analyse data in a single modelling framework.

**Fig. 2:**
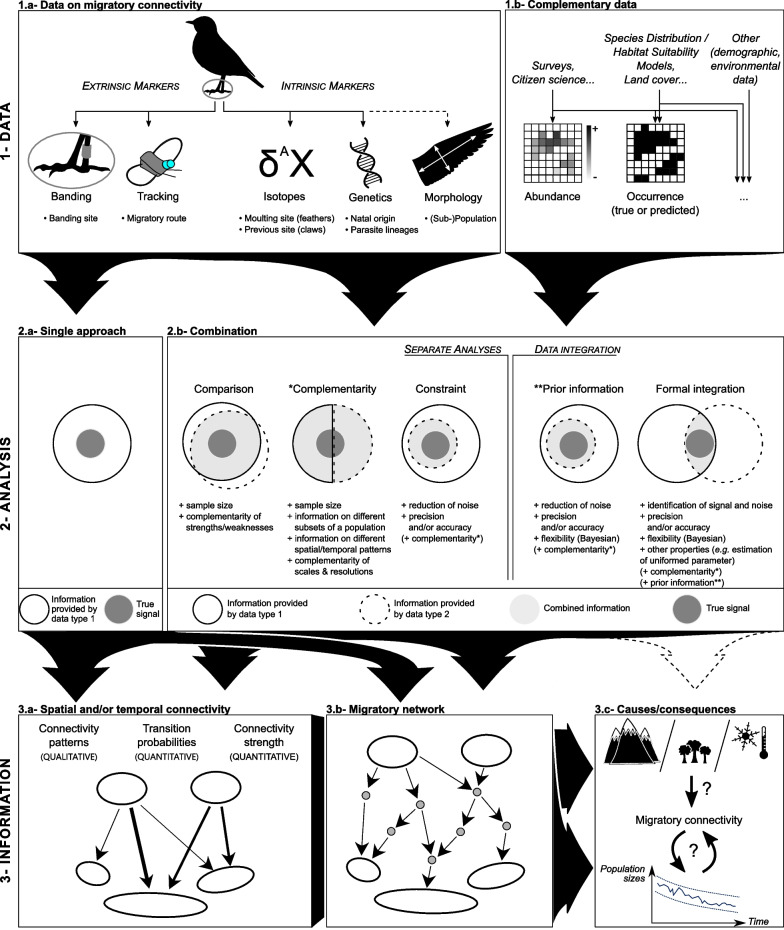
Conceptual framework of migratory connectivity studies. Large arrows show which data types are used in which analysis, and what information can be obtained from these. Various types of data provide more or less direct information on the movement of individuals between sites or populations (**1.a**), which can be used either alone (**2.a**) or in combination (**2.b**) to investigate migratory connectivity at different scales and resolutions (**3.a, b**). Complementary data such as species abundance or occurrence (**1.b**) is increasingly included in combination to improve the precision and/or accuracy of analyses. Recent studies build on the output of migratory connectivity studies (**3.a, b**) to investigate the evolutionary causes and consequences of this connectivity in relation to additional environmental variables or population dynamics (**3.c**). The possibility of integrating such additional data at the same level as other complementary data (**1.b**) needs further investigation in order to consider migratory connectivity in its functional and dynamic aspects rather than as a static description

### Separate analyses of different data types

Combining information from separate data analyses was the first form of data combination to appear in migratory connectivity studies [71, 91]. The benefits resulting from such combinations depend on the degree to which the different data contains the same information (Fig. 2.2).

Most often, studies compare the consistency of results derived from different data types (e.g. [29, 52]). This can be a way to increase sample size, particularly for species for which very few banded birds have been reencountered or few tracking devices fitted [51]. The comparison of different data types can also prove useful when one data type is insufficient to detect connectivity patterns. In the case of golden-winged warblers (*Vermivora chrysoptera*) and blue-winged warblers (*Vermivora cyanoptera*), tracking data revealed a stronger connectivity pattern than that obtained from genetic analyses, highlighting the unexpected low differentiation of previously used genetic markers [63].

Some studies focus on the complementarity of the information resulting from different types of data rather than simple consistency in results. Each data type can provide information on different subsets of a population. As pointed out by Finch et al. [53], band recoveries provide information on juveniles and individuals that failed their migration whereas tracking data is biased towards successful and heavier older birds. Different types of data can also provide information at different scales and resolutions. The resolution of tracking data can, for instance, serve as a means to check the accuracy of isotopic assignments [47]. In the case of genetic and isotope data, the complementary information they respectively supply about longitudinal and latitudinal location improved the resolution of spatial assignments in North America [71, 93].

Precise information obtained with one data type has also been used as a constraint in the analysis of another data type, e.g. by restricting the area of interest for spatial assignments [83]. This is appropriate for increasing the signal-to-noise ratio by avoiding the inclusion of irrelevant regions. Isotopic spatial assignments notably benefit from tracking data [29, 83] or knowledge about the distribution range of the studied species [29, 48, 82], which can be used to limit the final assignment area, improving the spatial resolution of analyses.

### Integrated modelling

Data integration sensu stricto is based on a model that inputs a variety of data that is analysed within a single framework. An integrated model can be seen as composed of multiple submodels—one for each type of data—which are combined in a core equation. If the different datasets contain independent information on a similar variable, then this variable appears in each of the corresponding submodels, and can thus be estimated from all these data at the same time. Schematically, the information from the various datasets is crossed, allowing for a better isolation (or “identification”) and estimation of that variable (Fig. 2.2). Although the foundation of integrated approaches in migratory connectivity studies was established in the first half of the 2000s [49], it really began to emerge in the last decade (Fig. 1). Most integrated connectivity analyses are conducted in Bayesian frameworks due to the inherent flexibility of the underlying algorithms (Box 2).

**Box 2:** An example of integrated modelling in migratory connectivity studies

The model of Korner-Nievergelt et al. [57] has served as a basis for multiple integrated analyses using Bayesian statistics in the past few years as it combines two classic types of data informing about migratory movements in a flexible framework. It therefore makes a perfect example to illustrate how various data can be integrated in migratory connectivity studies. This model combines reencounter data and tracking data of individuals banded/tagged in breeding areas $g \in \left[ {1;G} \right]$ and resighted/tracked in non-breeding destination $d \in \left[ {1;D} \right]$ to estimate the probabilities of movement—or transition probabilities—between breeding and non-breeding sites. Here, these transition probabilities are by definition the migratory connectivity parameters of interest.	
**1. Writing the likelihoods**	
The first step in Bayesian statistics is to formulate the *likelihood*, which represents the probability of having the observed data knowing a set of parameters. It boils down to expressing the data as a function of chosen *parameters* using statistical models:	
* Submodel #1: The probability of reencountering a banded individual* from breeding area $g$ in destination $d$, $P_{g,d}^{reenc}$, can be expressed as the conjunction of two events: “The individual moved from area $g$ to destination $d$ (transition probability $m_{g,d}$) AND could be observed in destination $d$ (reencounter probability $r_{d}$)”. Probabilistically, this can be translated into: $P_{g,d}^{reenc} = m_{g,d} \times r_{d}$, where we assume that the reencounter probability in destination $d$ is independent from the origin $g$ of the individual. The probability of not reencountering a banded individual in any destination is then: $P_{g,D + 1}^{reenc} = 1 - \mathop \sum \limits_{d = 1}^{D} \left( {{\text{m}}_{g,d} \times {\text{r}}_{d} } \right)$ so that probabilities sum to 1. The total number of banded individuals in breeding area $g$, $N_{g}^{reenc}$, can thus be related to the number of banded individuals from breeding area $g$ that were reencountered, $R_{g,d}$, (or not reencountered at all, $Q_{g}$) in each of the destination areas $d$ via a multinomial model: $\left( {R_{g,1:D} ,Q_{g} } \right) \sim Multinom\left( {P_{{g,1:\left( {D + 1} \right)}}^{reenc} ,N_{g}^{reenc} } \right)$. The final likelihood of the live-reencounter submodel is the product of these multinomial models for $g \in \left[ {1;G} \right]$. * Submodel #2: Tracking devices* give direct information about which destination $d$ an individual tagged in area $g$ moved to—if the recovery bias can be ignored for archival tags such as geolocators. In this case, the probability of tracking an individual from area $g$ to destination $d$ can be simply expressed as: “The individual moved from area $g$ to destination $d$ (transition probability $m_{g,d}$)”. Probabilistically, this can be translated into: $P_{g,d}^{track} = m_{g,d}$. Similar to the live-reencounter submodel, the total number of tracked individuals in breeding area $g$ for which the data could be retrieved, $N_{g}^{track} = \mathop \sum \limits_{d = 1}^{D} U_{g,d}$, can thus be related to the number of tracked individuals from breeding area $g$ that moved to each of the destination areas $d$, $U_{g,d}$, via a multinomial model: $U_{g,1:D} \sim Multinom\left( {P_{g,1:D}^{track} ,N_{g}^{track} } \right)$. The final likelihood of the tracking submodel is the product of these multinomial models for $g \in \left[ {1;G} \right]$.	
Since these two submodels share the same connectivity parameter $m_{g,d}$, they can be integrated by formulating a *joined likelihood*. If the two datasets are independent, the joined likelihood is equal to the multiplication of the likelihoods of all submodels.	
**2. Specifying the prior distributions**	
Bayesian models use the likelihood to update *prior distributions* in a Markov chain, which produces a *posterior distribution* of values for each parameter. Obtaining a distribution of values for the parameters, instead of a single value, is characteristic of the Bayesian approach. The second step to run a Bayesian model is thus to specify prior distributions for the parameters to estimate. In their model, Korner-Nievergelt et al. [57] chose to use non-informative flat priors: all parameters were assumed to be uniformly distributed between 0 and 1 (following a $Beta\left( {1,1} \right)$ model for $r_{d}$ and the multivariate equivalent $Dirichlet\left( {1, \ldots ,1} \right)$ for $m_{g,1:D}$). This means that the transition probabilities and the reencounter probabilities were allowed to converge towards any value between 0 and 1. However, prior distributions can be restricted to certain values only and thus act as a smooth constraint on the posterior distribution of the parameters. In other models, this has been a second entry door for data combinations, which has for instance been used to refine spatial assignments with abundance data or migratory directions inferred from banding data (e.g. [49, 94]).	
Following the same reasoning, new sub-models have been added to this structure to integrate isotope data, parasite data, or even take into account banding data with unknown numbers of banded birds or recovery biases for geolocators [12, 39, 40]. This flexibility makes the strength of Bayesian frameworks for data integration.	

Integrated models were first used to include various types of prior information, which act as a smooth constraint on the analysis parameters based on prior knowledge of the studied system [49]. This method allowed new types of data to be combined with common banding, tracking, isotope and genetic approaches to further increase the accuracy of results [49]. Data on breeding abundance is, for instance, integrated as a prior since the probability of originating from a population does not only depend on the frequency of the marker of each population of origin, but also on the relative size of these populations (e.g. [49, 82]). Other complementary data include knowledge of migratory directions based on banding data [48, 94] or population genetic structure [95], as well as information on species distribution [74, 96].

Studies that quantify migratory connectivity (i.e. where connectivity takes the form of an estimable parameter such as transition probability between sites, see Box 1) can fully integrate different data if the statistical models used to analyse the data share common parameters [57] (Box 2). The Bayesian framework is unequivocally preferred since it allows the straightforward combination of probabilistic models (see, however, [97], for another statistical framework), provided datasets provide information on the same population and are independent [57, 98]. By extending the structure of banding multi-state models to simultaneously analyse tracking data, Korner-Nievergelt et al. [57] significantly increased the precision of their connectivity estimates. Integrated analyses are more powerful than independent analyses in disentangling the intersecting information in datasets—the shared parameter(s)—from the data-specific uncertainty, allowing more information to be extracted from each dataset. Integrated models have been used to jointly analyse banding and tracking data [12, 39, 57], as well as combinations of banding, isotope and parasite data [40] or isotope and genetic data [74, 95, 98].

Additionally, integrated models have shown interesting results in other fields of ecology, such as the ability of integrated population models to estimate uninformed parameters [99]. This property originates from the fact that some data bears information on various processes altogether—e.g. survival, fecundity, and migration rates for population counts. These processes can be isolated and identified by crossing the entangled mass of information of population counts with the more specific information brought by other independent sources of data (e.g. breeding monitoring, capture-recapture). If the specific information brought by the other data allows isolation of all but one of the processes, this last process logically appears isolated and identifiable too. It would be reasonable to think that population counts for migratory species also bear information about migratory connectivity, which is supported by recent work modelling migratory connectivity directly from abundance data [89, 90], in the same way as the various connectivity data bear information about demographic rates (e.g. survival for banding data). Integrating population models and migratory connectivity models would be an interesting lead to follow to push further our understanding of the interplay between population dynamics and migratory connectivity. Such properties—associated with the wide range of possible data that integrated models can jointly analyse—suggest that they are among the most promising tools for migratory connectivity studies.

## Challenges of combining data in migratory connectivity studies

The diversity of available methods raises questions about which type of data and/or combination should be favoured, and how to optimise their use. The performance of different analyses is generally compared based on the precision of spatial assignments [100] or connectivity estimates [57]. The accuracy of results has also been assessed by evaluating the assignment of samples or individuals of known origin (e.g. [95, 97]) or by comparing the results to high-resolution tracking data (e.g. [47]).

### What data to prioritise?

To our knowledge, no study has provided an explicit answer as to which data should be favoured in migratory connectivity studies. How to optimise the sampling design to get the best description of connectivity patterns needs to be addressed, as collecting data for more species to build connectivity atlases will be a costly and time-consuming process [82].

Since migration leads birds to occupy different places at different times, often covering large geographic areas, it may be worth questioning where and when data should be collected to draw the best picture of connectivity patterns. Some data may be more informative and have a greater signal-to-noise ratio if collected during a specific season. For example, geolocator data shows higher latitudinal accuracy at higher latitudes, so tagging individuals on non-breeding grounds at low latitudes and collecting data on their movements towards the breeding grounds at high latitudes should be more informative than tagging birds on the breeding grounds and tracking them to their non-breeding grounds [101]. Furthermore, quantified estimates of migratory connectivity are sensitive to sampling designs and can be biased if trying to make an inference at a broader extent than the sampled area [69]. Some studies have started providing guidelines to understand the effects of the sampling design on connectivity estimates and adapt it to the study system [102]. More generally, applying statistically randomised sampling designs across the migratory cycle may be a promising direction to improve estimation [68]. Most studies rely solely on data collected during either the breeding or non-breeding season, or autumn or spring migration (e.g. [39, 103]). Yet study robustness may benefit from sampling at breeding, non-breeding and stopover sites, which may help avoid sampling bias, especially when populations mix between seasons (low connectivity strength, see Box 1; [8]).

As for the type of data to collect, all approaches used to track birds balance the scale and resolution of data in different ways. Tracking data is increasingly used to get a picture of migratory connectivity at high resolution, even at the scale of the migration route [54], which also holds promise for behavioural studies [28, 31]. Yet the ability of isotope data to describe connectivity patterns for hundreds of individuals across several species [82] and even over long time periods [85] is incomparable regarding the scale and representativeness of results. The complementarity of these two data types illustrates why no one today advocates for the use of a single data type. This is further explained by the restrictions and advantages of the different methods to collect data, which depend on the characteristics of the species. For instance, tracking technologies are usually limited by the size and weight of the individuals, although the progress of telemetry, light loggers, and even GPS tags now allows tracking of passerines [104]. Isotope data is mainly usable for species that grow their tissues in areas with clear isotopic signatures or gradients, as it is the case for species breeding in North America [82]. Similarly, genetic data is highly informative if breeding populations have a strong genetic identity that can be characterised [63], which is more probable for species with low breeding dispersal. Finally, bird-banding can be used for every species as long as the detection and capture probabilities are not too low and that large-scale effort can be put into band reading.

The fact that, currently, no approach is clearly superior to another instead shifts the question to whether all data can be combined, and whether certain combinations are more effective than others. For instance, the high longitudinal resolution of geolocator data would pair well with the good latitudinal resolution of hydrogen isoscapes in North America or Europe [82, 101]. This question has become even more important through the years as the combination of connectivity and more diverse complementary data, such as spatial occurrence, have proven to significantly improve the scale and resolution of the migration patterns we can draw [105]. Consideration needs to be given to the constraints associated with differences in scale and the representativeness of the data to be combined. Despite being put forward as a strength of combining data, mismatches in scale and sampling bias need to be handled with care to avoid further skewing results [106]. It is all the more important to consider these questions as new data combinations may appear with the development of integrated approaches.

### How to optimise the combined analysis of different data types?

Investigating the performance of independent versus combined analyses suggests that not all combinations are relevant. In their study, Ruegg et al. [74] showed that the precision obtained with genetic data alone was almost equivalent to that obtained when combining genetic and isotope data with predictions of habitat suitability models. More strikingly, Hobson et al. [82] and Reese et al. [107] showed that including abundance data can sometimes worsen assignments. Indeed, abundance data can be particularly spurious when populations occur in patches because models then tend to neglect information about individuals that come from low density regions [103].

A proper understanding of what data is worth combining, and how to do it, requires understanding how each data type can contribute to the final results. This contribution should not necessarily be equal due to differences in the quality and quantity of the data. By quantifying the overlap between the posterior distribution of independent models with the posterior distribution of the integrated model, Korner-Nievergelt et al. [57] and Von Rönn et al. [40] observed that the contribution of each data type to the estimation of a given connectivity parameter was related with the actual amount of data they had for this region. In other words, for each parameter, the integrated models relied more on the most abundant type of data that could inform on it. Korner-Nievergelt et al. [57] stressed the need to improve current models to account for sampling effort and thus release the strain exerted by the most abundant data, which is not necessarily more informative than a smaller dataset sampling rigorously the study system.

The first way to optimise combined analyses would be to investigate which combination method performs best given the data at hand. As shown for species distribution models, the combination method can indeed affect the accuracy of the results [108]. Different combination methods give different weights to the various data (see Fig. 2.2), so the choice of a method is a first way to account for differences in quality and quantity of the data [108]. Future studies would thus benefit from a general comparison of the different types of combination that have been used so far, as well as a more specific assessment of how well various combination methods perform for their study system.

Once a combination method has been chosen, it is possible to adjust it to further control the contribution of the data to the results. Various attempts have been made to weight contributions of multiple types of data within a model according to the confidence researchers had in the different datasets. For instance, Ruegg et al. [74] and Rundel et al. [98] raised the probability of origin obtained for each data type to different powers when combining them using Bayes’ rule, tuning the parameters to optimise the spatial assignments of individuals of known origin in a cross-validation procedure. However, as stated in these two studies, such weighting may differ between groups of birds and be dataset dependent. The added value of weighting data has been highlighted using optimisation methods. Rushing et al. [100] found that for some species, correct weighting of isotope and abundance data decreased the surface and error of assignment, but incorrect weighting could increase both of these, or cancel out an increase in precision by a major increase in error rate (or vice versa). Combining data therefore can give less accurate results than the analysis of a single data type if not carefully tested using known source samples. More work is thus needed to assess whether the contribution of each data type should be modified and how to do this objectively [106].

### Which approaches to integrate data across the migratory cycle?

Migratory connectivity has important implications for population dynamics, and is crucial to predicting the impact of climate change on migratory birds [19] and devising conservation measures [17]. Indeed, migratory species are particularly sensitive to site degradation and to localised events that may affect the survival or reproductive success of individuals, either directly or through alterations to the body condition or phenology of individuals that carry over to the subsequent seasons [7, 109]. The consequences of such events that affect only part of the migratory populations cannot be understood without a proper knowledge of where individuals originate from and where they go. Investigating the relationships between migratory connectivity and demographic and environmental processes has thus become a natural direction for recent studies about migratory species. For instance, information on migratory connectivity has been combined with demographic and environmental data using demographic models to understand how connectivity affects population counts and demographic parameters [110]. Conversely, migratory connectivity has also been investigated in light of network theory to understand how changes in populations or sites might affect the whole migratory network [15, 111]. However, information about migratory connectivity is still lacking for the vast majority of species and the methods to include such data into population dynamics analyses are still developing.

Current demographic models generally consider migratory connectivity as a static input to estimate the degree to which changes in a given region may affect population dynamics rather than as a variable ecological process [110, 112]. To our knowledge, connectivity has only been thought of as dynamic in theoretical network models where it can be estimated as an equilibrium output affected by changes in the migratory network [15, 113]. Such dynamics need to be understood now that we are able to more clearly describe patterns of migratory connectivity. The integration of connectivity and population data is likely to bring insights on the effects of connectivity on population and migratory network dynamics—and potentially the inverse relationship. This data integration shows promise to further advance the performance of full annual-cycle population models that consider events in the breeding, non-breeding and migration periods, and their consequences on population dynamics [10, 114]. The development of such bridges between migratory connectivity, environmental, and population dynamics tools has great potential to improve our ability to conserve species.

## Conclusion

Technical and methodological advances over recent decades have greatly improved the quality, reliability and accessibility of the bird movement data that underpins migratory connectivity patterns. In parallel, the diversification of methods to combine data has further boosted the precision and accuracy of patterns and estimates, as well as their spatio-temporal resolution and scale. The various methods for tracking birds through space, and for combining this data with complementary information, are two important dimensions to be considered in the toolbox to investigate migratory connectivity. Data combinations have not been systematically used in previous studies mainly because only one dataset had been collected for the species of interest, or because only one type of data was informative enough for the study. Now that large-scale data collection must be carried out to build the long-awaited connectivity atlases [82], the potential of combined analyses should be kept in mind. However, the diversity of methodologies that now compose this toolbox pose a number of questions. No approach currently seems to outperform the others, making it hard to navigate through the jungle of methodologies. In this context, further work is needed to assess the performance of these methodologies, to determine where and when connectivity data should be collected and how to get the best of data combinations. Answering these questions is even more important as migratory connectivity starts to be considered in a broader range of ecological questions. New data are susceptible to be analysed along with migratory connectivity to further understand the behaviour of migratory species, their population dynamics, evolutionary history, and sensitivity to global changes. Notably, data integration has the potential to provide insights into the functional aspects of migratory connectivity, allowing an understanding of how connectivity affects the response of migratory populations to environmental changes and localised pressures, and how it may in turn be affected by these changes and pressures. Data integration is therefore likely to be a major tool for opening up the field of migratory connectivity in the coming years.

### Supplementary Information

**Additional file 1**. Filtering of publications from the Web of Science: methodology for searching and analysing the Web of Science database for what concerns migratory connectivity studies.

## Data Availability

Certain data included herein are derived from Clarivate Web of Science. © Copyright Clarivate 2023. All rights reserved. https://www.webofscience.com. Web of Science and Clarivate are trademarks of their respective owners and used herein with permission.
