# Supplementary material for: A synthesis of recent tools and perspectives in migratory connectivity studies
Source: Mov Ecol. 2023 Oct 27;11:69. doi: 10.1186/s40462-023-00388-z (PMC10605477; doi:10.1186/s40462-023-00388-z)
Supplement: Supplementary file 1 — Additional file 1. Filtering of publications from the Web of Science: methodology for searching and analysing the Web of Science database for what concerns migratory connectivity studies. [file 40462_2023_388_MOESM1_ESM.docx]

**Additional file 1**

***Title:*** A synthesis of recent tools and perspectives in migratory connectivity studies

***Authors:*** Killian A. Grégory^1,2,3^*, Charlotte Francesiaz^4^, Frédéric Jiguet^2^, Aurélien Besnard^5^

***Affiliations:***

^1^*Master de Biologie, École Normale Supérieure de Lyon, Université Claude Bernard Lyon 1, Université de Lyon, Lyon, France*

^2^*CESCO, MNHN-CNRS-Sorbonne Université, Paris, France.*

^3^*CEFE, Univ Montpellier, CNRS, EPHE, IRD, Montpellier, France*

^4^*OFB, DRAS, Juvignac, France.*

^5^*CEFE, Univ Montpellier, CNRS, EPHE-PSL University, IRD, Montpellier, France.*

*corresponding author: Killian A. Grégory, [killian.gregory@ens-lyon.fr](mailto:killian.gregory@ens-lyon.fr)

***Keywords:*** migration patterns; transition probabilities; data pooling; integrated modelling; population dynamics; movement ecology

**A- Filtering of publications from the Web of Science (Figure 1)**

Following operations were performed using Web of Science search tools (© Copyright Clarivate 2023, all rights reserved) and R v.4.0.2 (R Development Core Team, 2020).

1. [Web of Science] **Base**: All publications containing “migrat* connectivity” in All Fields of the Web of Science Core Collection (last research 02/11/2023).
2. [R] **Filter the base**:

- Remove duplicates
- Remove papers with missing abstract
- Keep publications containing “migrat* connectivity” in Title, Abstract or Author Keywords
- Remove publications about neurones, ungulates, bats, turtles, tortoises, fishes, sharks and insects (keep when “bird” or “*streptopelia*” appear and finish filtering manually).

1. [R] **Combinations**: Base publications using/discussing at least 2 different types of data to investigate migratory connectivity, identified using specific search patterns (not words):

- Bird banding:
  - live reencounter
  - live-reencounter
  - band
  - recovery
  - recoveries
  - recapture
  - ring
  - resight
- Tracking:
  - tracking
  - gps
  - gls
  - gsm
  - geolocat
  - argos
  - radio
  - telemetry
  - + manual check and selection of papers with “ track” or “ tag” (3 concerned)
- Genetics:
  - genetic
  - genomic
  - microsatellite
  - snp
  - dna
- Isotopes:
  - isotop
  - hydrogen
  - carbon
  - nitrogen
  - sulfur
- Other:
  - abundance
  - sdm
  - species distribution
  - habitat suitability
  - occurrence
  - citizen science
  - morpholog
  - parasit
  - forest cover

1. [R] **Integration**: Combination publications corresponding to data integration *s.s.*, identified using specific search patterns (not words):

- Integration:
  - integrat
  - pairing
  - joint
  - prior
- Manual filtering of the ~35 obtained publications since integration keywords are used in various contexts, most of which are irrelevant here.

1. [R] **Unclassifiable**: Publications that did not fit in the previous categories (*i.e.* no search pattern was detected: 7% of the total number of publications). These consisted in theoretical reviews that did not mention any data, purely modelling studies, and studies in which migratory connectivity was too marginal to be mentioned in the title, abstract, or key-words.

*NB:* The Web of Science does not contain all existing articles on migratory connectivity; however, for the sake of repeatability, we did not add identified missing publications. Showed trends are assumed to be representative as no segregation is expected in the inclusion of publications in the Web of Science between those using/discussing a single type of data, a combination, or an integration.
